# Development of a diagnostic model for MASLD and identification of daidzein as the potential drug using bioinformatics analysis and experiments

**DOI:** 10.3389/fimmu.2025.1698740

**Published:** 2025-10-22

**Authors:** Tao Wang, Hao Zhang, Kaixia Wang, Chunxue Liu, Nan Kong, Luocheng Zhou, Lihong Qu

**Affiliations:** ^1^ Department of Infectious Diseases, Shanghai East Hospital, School of Medicine, Tongji University, Shanghai, China; ^2^ Department of Endocrinology, Shanghai East Hospital, School of Medicine, Tongji University, Shanghai, China

**Keywords:** metabolic dysfunction-associated steatotic liver disease, machine learning, SHAP, ENO3, daidzein

## Abstract

**Background:**

Metabolic dysfunction-associated steatotic liver disease (MASLD) is now the predominant chronic liver disease globally, yet effective therapeutic strategies remain elusive.

**Methods:**

MASLD-related datasets were download from GEO. Subsequently, genes associated with MASLD were found through the intersection of differentially expressed genes and WGCNA. Then, key candidate genes were further screened using 113 machine learning algorithms and their diagnostic value was evaluated using ROC curve analysis across multiple datasets. Genes are then screened by Shapley Additive exPlanations (SHAP) analysis. Molecular docking (MD) and molecular dynamics simulations (MDS) were employed to validate the interaction between Daidzein and Enolase 3 (*ENO3*). Finally, an *in vitro* fatty liver cell model was constructed to validate the “Enrichr” platform to identify poteitial drugs for MASLD.

**Results:**

62 MASLD-DEGs were finally identified. The optimal predictive model for MASLD was the 17-gene signature (*IGFBP1*, *ENO3*, *SOCS2*, *GADD45G*, *NR4A2*, *RTP4*, *RAB26*, *CRYAA*, *PPP1R3C*,*MCAM*, *IL6*, *IER3*, *RTP3*, *NR4A1*, *CCL5*, *FOS*, *JUNB)* selected through combined glmBoost+GBM algorithms, which was demonstrated robust predictive performance. SHAP analysis suggested that ENO3 may be the most prominent genes associated with MASLD severity. More importantly, we measured the effect of daidzein on improving lipid accumulation *in vitro* model.

**Conclusion:**

We developed a predictive model for MASLD and identified *ENO3* as a key predictive gene. Furthermore, we discovered that daidzein may serve as a potential therapeutic agent for MASLD. Through *in vitro* studies, we further confirmed that daidzein alleviates lipid deposition and improves MASLD by modulating the ENO3/PPAR signaling pathway.

## 1 Introduction

Metabolic associated fatty liver disease (MAFLD) is an enormously prevalent chronic hepatic condition that constitute a significant portion of cases worldwide, seriously jeopardizing human health and public health resources (1). Metabolic dysfunction-associated steatotic liver disease (MASLD), which replaces the term MAFLD, encompasses a clinical spectrum progressing from simple fatty liver (MAFL) to steatohepatitis (MASH), and may further advance to serious complications such as fibrosis, cirrhosis, and hepatocellular carcinoma (2). A Meta analysis (3) showed that the global prevalence of MASLD was 30.05%, and the prevalence of MASLD in China was even higher, reaching 32.9%, and showing a rapid growth trend. According to statistics, in 2020, the number of MASH patients in China is about 38.7 million, and it is expected to reach 46 million by 2025 and increase to 55 million by 2030 (4).

Hepatic histologic assessment is often used as a surrogate endpoint in MASLD clinical trials (5). However, liver biopsy is an invasive procedure, costly, and associated with postoperative adverse effects such as infection, bleeding, and pain, which are generally difficult for MASLD patients to accept (6). More importantly, liver biopsy can only assess very small liver samples, while limited liver sampling may lead to significant errors in determining diagnosis, disease staging and longitudinal evolution given the known spatial heterogeneity of diffuse liver disease (7). So, noninvasive research for diagnosis and assessment of response to therapy is of particular importance.

Development of effective drugs to treat MASH is a major concern for the general public. Thankfully, in March 2024, the U.S. FDA approved Resmetirom for the treatment of adult MASH patients with stage F2-3, but its adverse effects and high price have limited the development of the drug (8).

Machine Learning (ML), as an important branch of Artificial Intelligence, through the learning and analysis of massive data, can automatically extract the features and patterns in the data (9), realize the automated consultation and preciseness assessment of imaging for diseases, and reveal the links between genes and disorders, which not only raises the accuracy and efficiency of diagnosis (10), but also provides a scientific basis for personalized medicine (11).

Daidzein, also known as soy isoflavone, is derived from soybeans and legumes and is a natural isoflavone compound (12). Due to their ability to regulate lipid metabolism and their antioxidant, anti-inflammatory, and anti-cancer effects, they are widely used in the treatment of various diseases.

In our study, we pressed in multiple MASLD cohorts from the Gene Expression Omnibus (GEO) database, constructed MASLD prediction models by 113 machine learning combinations, screened for the best predicted genes using Shapley Additive exPlanations (SHAP) analysis, and most importantly, identified daidzein, an effective drug for MASLD, and validated it at the cellular level.

## 2 Methods and materials

### 2.1 Data collection and preparation

We analyzed transcriptomic data from 434 patients with MASLD and 132 healthy controls, sourced from 8 independent datasets in the Gene Expression Omnibus (GEO) database, including GSE24807,GSE33814, GSE63067, GSE89632, GSE48452, GSE66676, GSE126848, GSE130970 and GSE135251. The “combat” algorithm in the R package “sva” (13) was used to normalize and merge GSE24807,GSE63067, GSE89632 and GSE33814 into a training set and “normalizeBetweenArrays” algorithm from the “limma” package was utilized for data correction (14). GSE12684, GSE130970, GSE135251 and GSE48452 were 4 independent validation cohorts. GSE61260 and GSE66676 were used as external validation cohorts. Principal component analysis (PCA) and boxplots were further validated for quality control. Detailed information such as platform, samples and GSE series on these datasets is shown in **Table 1**.

**Table 1:** Basic information of GEO datasets used in the study.

GSE series	Samples	Platform	Group
GSE24807	12 MASLD and 5 controls	GPL2895	Training cohort
GSE33814	31 MASLD and 13 controls	GPL6884	Training cohort
GSE63067	11 MASLD and 7 controls	GPL570	Training cohort
GSE89632	39 MASLD and 24 controls	GPL14951	Training cohort
GSE48452	32 MASLD and 41 controls	GPL11532	Validation cohort
GSE126848	31 MASLD and 26 controls	GPL18573	Validation cohort
GSE135251	206 MASLD and 10 controls	GPL18573	Validation cohort
GSE130970	72 MASLD and 6 controls	GPL16971	Validation cohort
GSE61260	47 MASLD and 38 controls	GPL11532	External validation cohort
GSE66676	34 MASLD and 33 controls	GPL6244	External validation cohort

### 2.2 Identification of MASLD related differentially expressed genes

The “limma” package (14) in R software was used to identify the DEGs in the training set, with standards of |log2 FC| > 0.585 and adj *P*-value <0.05. Next, we constructed a co-expression network for MASLD using Weighted Gene Co-expression Network Analysis (WGCNA) (15) to find the most relevant modules for MASLD for subsequent analysis. Finally, genes in the module that intersect with DEGs are then considered MASLD-DEGs.

### 2.3 Enrichment analysis of MASLD-DEGs

We used the clusterProfiler (16) software package for Gene Ontology(GO) analysis to reveal MASLD-DEGs in biological processes(BP), cellular components(CC) and molecular functions(MF). In addition, GO, Kyoto Encyclopedia of Genes and Genomes (KEGG) and Disease Ontology (DO) analysis were performed to find the molecular mechanism behind MASLD-DEGs.

### 2.4 Machine learning algorithms

To construct the best model, we use a combination of 113 permutations of 12 machine learning algorithms including LASSO, Ridge, Stepglm, XGBoost, Linear Discriminant Analysis (LDA), Generalized Linear Model Boost (glmBoost), Elasticity Networks (Enet), Partial Least Squares Regression for Generalized Linear Models (plsRglm), Generalized Boosted Regression Modeling (GBM), Random Forest (RF), Simple Bayes, and Support Vector Machines (SVM). As mentioned above, we combine GSE24807,GSE63067, GSE89632 and GSE33814 as training set while using GSE126848, GSE130970, GSE135251 and GSE48452 as validation set respectively. To obtain the optimal model, we employ an ensemble learning strategy that performs weighted averaging of predictions from various algorithms. This approach enhances model robustness and reduces the risk of overfitting. Concurrently, k-fold cross-validation is utilized to ensure the model demonstrates consistent performance across different validation sets.We used the AUC value of the validation and training sets and the number of genes included in the model as selection criteria for the best model.

### 2.5 SHAP model for the diagnosis of MASLD

SHAP is a method for interpreting the prediction results of machine learning models, and its goal is to compute, for each prediction made by the model, a value for the contribution of each input feature to the prediction result (i.e., the SHAP value) (17). The study employed repeated five-fold cross-validation, dividing the training dataset into five equally sized subsets. In each cross-validation cycle, four folds served as the training set, while the fifth fold functioned as the validation set to assess model performance.This value provides a clear indication of which features are most critical for a particular prediction outcome and whether they have a positive or negative impact on the prediction outcome. The core strength of the SHAP method is its ability to provide both local interpretability for individual prediction outcomes and global interpretability for the overall decision-making mechanism of the model (18, 19).

### 2.6 Characterization of potential anti-MASLD drugs

We used the Drug Signature Database (DSigDB) in the Enrichr web platform (https://amp.pharm.mssm.edu/enrichr/) based on the expression of 8 genes.

### 2.7 Molecular docking analysis

The 2D structure of the small-molecule ligand was obtained from the PubChem database (http://pubchem.ncbi.nlm.nih.gov/) and converted into a 3D structure using Chem Office software, followed by saving in MOL2 format. For the protein target, a high-resolution crystal structure was selected from the RCSB PDB database (http://www.rcsb.org/), then processed in PyMOL to remove water molecules and phosphate groups, yielding a refined PDB file. Then, use Autodock preprocessing to process the structures of proteins and small molecules, ultimately obtaining the optimal conformation for molecular simulation. Finally, PyMOL and Discovery Studio 2019 were employed to visualize and analyze the 2D/3D interactions between the ligand and key protein residues (20).

### 2.8 Molecular dynamics simulation

This study employed Gromacs 2022 (21) for molecular dynamics simulations. The protein force field was set to AMBER14SB, while the ligand force field utilized GAFF2, with parameters generated by the pdb2gmx tool and the AutoFF web server. The system was solvated in a cubic TIP3P water box with a dimension of 1 nm and neutralized by adding ions. Long-range electrostatic interactions were treated using the Particle Mesh Ewald (PME) method with a cutoff radius of 1 nm. Bond constraints were applied via the SHAKE algorithm with an integration time step of 1 fs. Prior to simulation, the system underwent energy minimization involving 3000 steps of steepest descent followed by 2000 steps of conjugate gradient minimization. A molecular dynamics simulation was performed under the NPT ensemble at 310 K and constant pressure for a duration of 100 ns. During the simulation, the following properties were calculated: root mean square deviation (RMSD), root mean square fluctuation (RMSF), number of hydrogen bonds (HBonds), radius of gyration (Rg), and solvent accessible surface area (SASA) (22).

### 2.9 Primary hepatocyte isolation

Primary hepatocytes were isolated from the livers of 6- to 8-week-old male mice. After perfusing the mouse liver with buffer solution via the portal vein and dissecting it, liver was placed in collagenase for digestion, filtered through a 70 μm filter to remove incompletely digested tissue fragments, and the resulting cell suspension was centrifuged at 4°C, 50 g, for 5 min. The supernatant was discarded, and the cell pellet was collected. The cell suspension was further purified by gradient centrifugation to isolate primary mouse hepatocytes, which were then counted and seeded for further culture (23).

### 2.10 *In vitro* model of MASLD

Primary hepatocytes were cultured in in high-glucose Dulbecco’s Modified Eagle Medium (DMEM) supplemented with 10% fetal bovine serum (FBS) and 1% mixed antibiotics, maintained in a constant temperature incubator at 37°C with a 5% CO_2_ atmosphere. To construct the MASLD model *in vivo*, primary hepatocytes were treated with complete culture medium containing the indicated concentrations of 0.33 mM palmitic acid (PA) and 0.66 mM oleic acid (OA) (LRB-X3, Kunchuang, Xian, China) for 24 h (24).

### 2.11 Cell counting Kit-8 assay

Cells were seeded at a density of 5.0×10³ cells per well in a 96-well plate. Cell viability was determined using the CCK-8 assay kit (Meilunbio, MA0218-1, China). Add 100 μL of culture medium and 10 μL of CCK-8 reagent to each well, and incubate for no more than 4 h. Subsequently, measure the absorbance of each well at a wavelength of 450 nm.

### 2.12 Cellular oil Red O staining

Primary hepatocytes were plated inoculated in 24-well plates and treated with 1 mM PO (0.66 mM OA + 0.33 mM PA) for 24 h in the presence or absence of low-dose Daidzein (50 μM) and high-dose Daidzein (100 μM). Cells were harvested and washed twice with phosphate buffered saline (PBS), followed by fixation with 4% neutral paraformaldehyde for 10 min. At the end of fixation, it was then washed 3 times with PBS, followed by immersion in 60% isopropanol for 15 s, and then stained for 10 min using Oil Red O working solution (60 % oil red O dye and 40 % distilled water). At the end of staining the cells were washed well using distilled water and then the nuclei were stained with hematoxylin, followed by observation of the cells under a light microscope.

### 2.13 Bodipy 493/503 staining

Cells were taken out of the incubator, washed twice with PBS, fixed by adding 4% paraformaldehyde for 10–30 min, and washed again 3 times with PBS. Bodipy 493/503 lipid dye (GLPBIO, GC42959, California, USA) was prepared at a final concentration of 2 μM in PBS. The 2 μM Bodipy 493/503 working solution was co-incubated with the cells at room temperature and protected from light for 15–30 min and then washed 3 times with PBS, followed by the addition of 4’,6-diamidino-2-phenylindole (DAPI) staining solution and then incubated for 5 min and protected from light and then washed 3 times with PBS before being imaged in a fluorescence microscope (25).

### 2.14 Cellular lipid content measurement

Primary hepatocytes were inoculated in 6-well plates (2×10^6^ cells per well). Then, the cells were co-cultured with daidzein (50, 100 μM) and PO for 24 h. Finally, triglyceride (TG) and total cholesterol (TC) levels were measured according to the instructions in the APPLYGEN test kit.

### 2.15 Quantitative real-time PCR analysis

A total mRNA of cultured cells was isolated using TRIzol reagent (Bioteke Corporation, RP40002), and synthesized into cDNA with a Reverse Transcription Master kit (Vazyme, R222-01). QRT-PCR was performed by using ChamQ SYBR qPCR Master Mix (Vazyme, Q311-02). The expression in control normalized the mRNA levels. The sequences of primer used in this study are displayed in **Table 2**.

**Table 2:** The sequences of primer used in this study.

Gene	sequence 5’ to 3’ forward	sequence 5’ to 3’ reverse
*ENO3*	ACAAAGCACGATACCTGGGG	GCGATGTGTCGGTAGAGAGG
*IGFBP1*	GCTGGATAGCTTCCACCTCATG	TCCATTTCTTGAGGTCAGTGATCTC
*SOCS2*	AGTTCGCATTCAGACTACCTACT	TGGTACTCAATCCGCAGGTTAG
*GADD45G*	AGAAGTTCGCGGCCAGGATA	GGACTTTGGCGGACTCGTAG
*NR4A2*	AAACTGCCCAGTGGACAAGCGT	GCTCTTCGGTTTCGAGGGCAAA
*RAB26*	GTCTGCTGGTGCGATTCAAG	GCATGGGTAACACTGCGGA
*RTP4*	ACATGGACGCTGAAGTTGGAT	TACGTGTGGCACAGAATCTGC
*CRYAA*	GGTGCTGGACTCTGGAATCT	ACTCACGGGAAATGTAGCCA
*β-actin*	GACAGGATGCAGAAGGAGAT	GAGGCCAGGATGGAGC

### 2.16 Western blot analysis

Samples were treated with RIPA buffer containing phosphatase and protease inhibitors, homogenized, and centrifuged, followed by a 15 min resting period. Protein concentration was then determined using the Bicinchoninic Acid (BCA) assay. Following electrophoresis, proteins were transferred onto a polyvinylidene difluoride (PVDF) membrane. The membranes were blocked for one hour using 5% skimmed milk powder and then incubated with primary antibodies at 4°C overnight. After washing with Tris-buffered saline with Tween 20 (TBST) the next day, the membranes were incubated with horseradish peroxidase (HRP)-conjugated secondary antibodies for one hour at room temperature. Protein detection was performed using equal volumes of enhanced chemiluminescence (ECL) solutions A and B (catalog number: U10012). Primary antibodies were shown in **Table 3**.

**Table 3:** Antibodies used for western blot.

Antibody	Source	Manufacturer
ENO3	Rabbit	Proteintech; 55234-1-AP
PPARα	Rabbit	Abcam; ab126285
PPARγ	Rabbit	Cell Signaling Technology; 2443S
PPARD	Rabbit	Proteintech; 28053-1-AP
FASN	Rabbit	Cell Signaling Technology; 3180S
SCD1	Rabbit	Cell Signaling Technology; 2794
CPT1A	Rabbit	Cell Signaling Technology; 12252
CD36	Rabbit	Cell Signaling Technology; 28109S
β-actin	Rabbit	ABGENT; AP14779b

### 2.17 Statistical analysis

All graphical analyses were conducted using R software (version 4.2.3). Normality of the data was assessed using the Student’s t-test, and correlation analyses were performed using Spearman’s correlation test. All statistical results were considered significant at *P*-values < 0.05, unless otherwise specified.

## 3 Results

### 3.1 Identification of MASLD-DEGs

**Figure 1:**
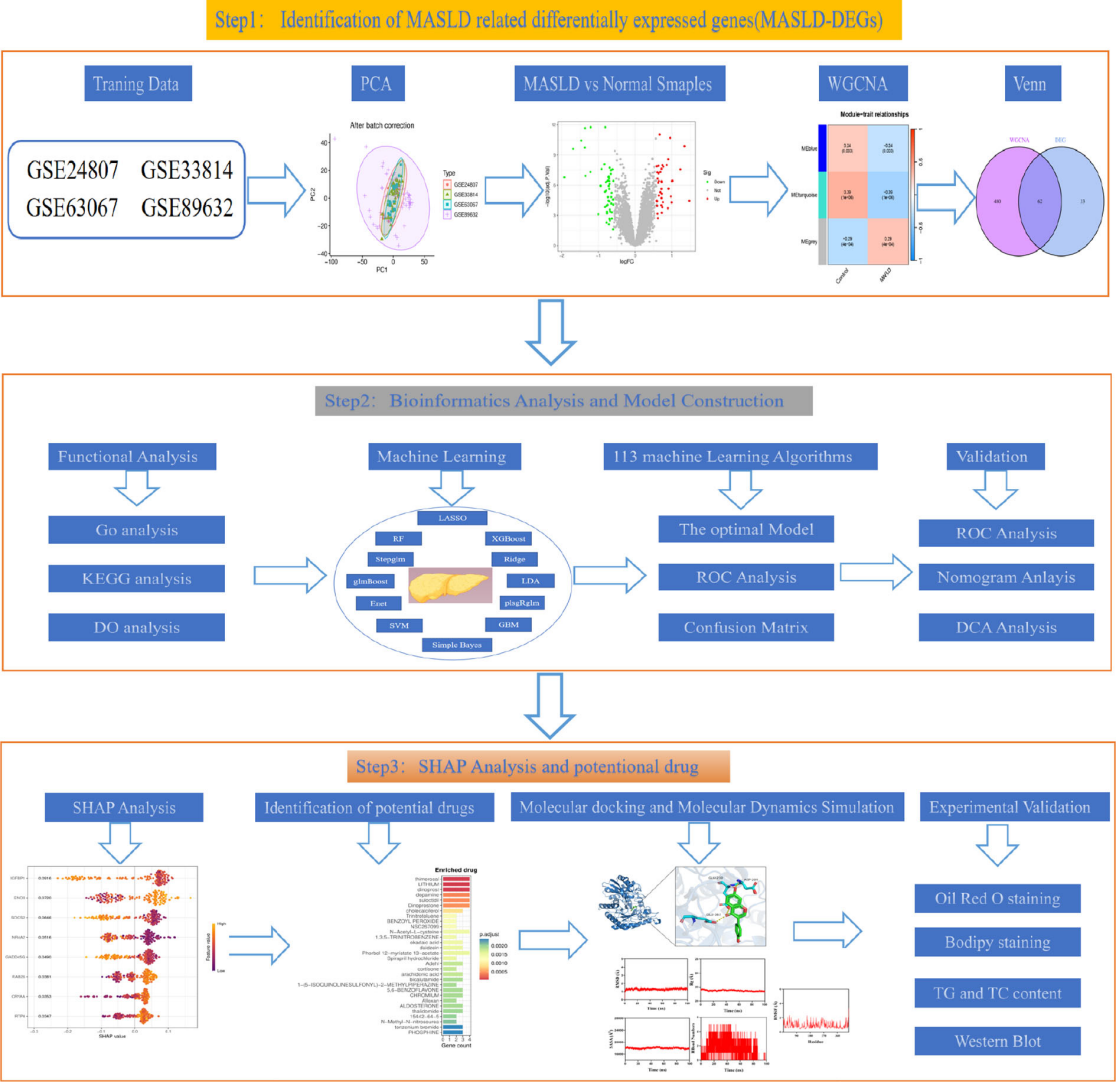
The flowchart of the manuscript.

**Figure 2:**
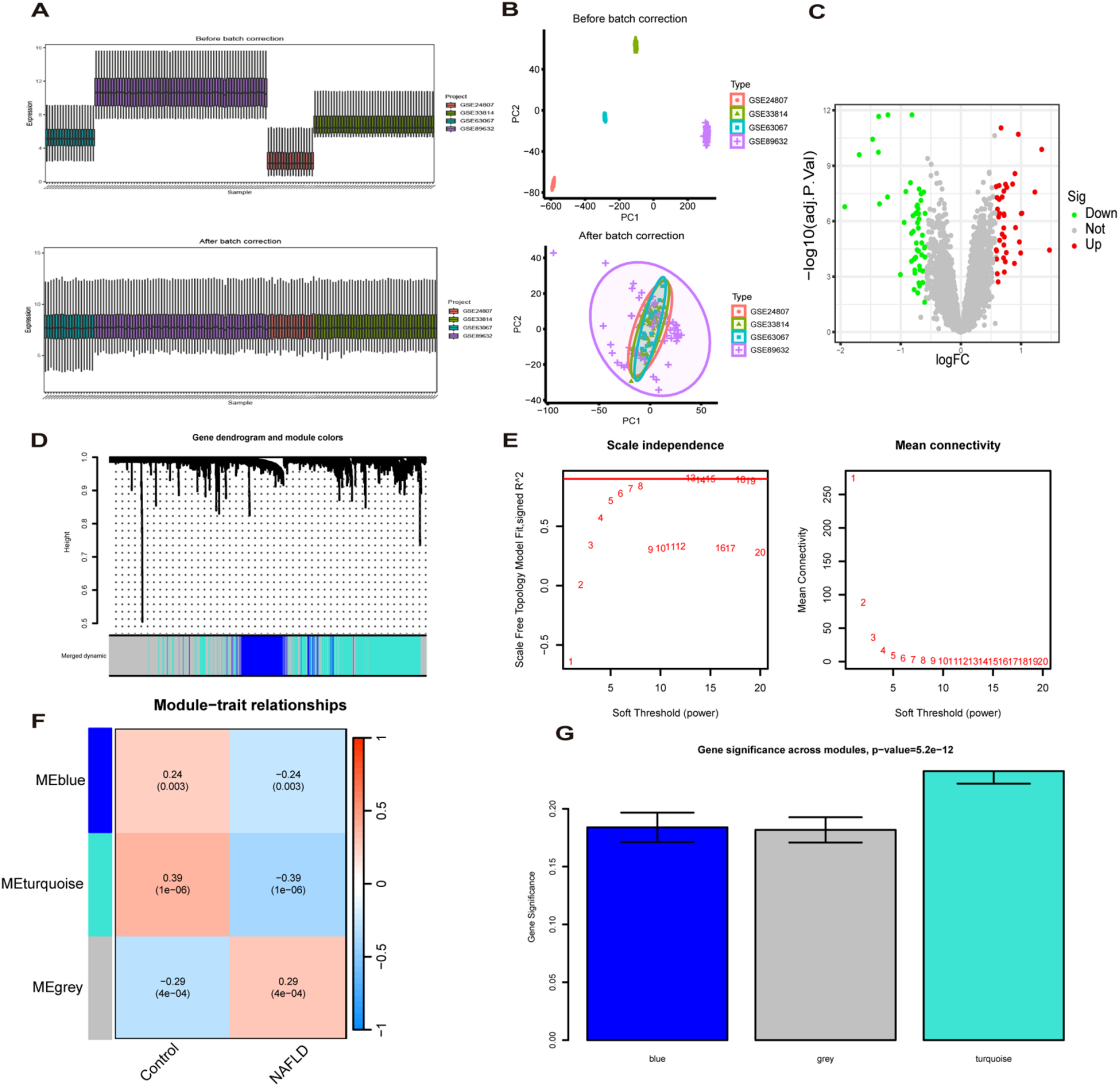
Recognition of MASLD related differentially expressed genes(MASLD-DEGs). **(A, B)** Box plots **(A)** and PCA plots **(B)** before and after normalization. **(C)** The Volcano plot shows differentially expressed genes (DEGs) in normal and MASLD samples. **(D)** Gene dendrogram and module colors. **(E)** The determination of soft-thresholding power. **(F)** Relationship between gene modules and traits. **(G)** Identification of the modules most relevant to MASLD progression.

**Figure 3:**
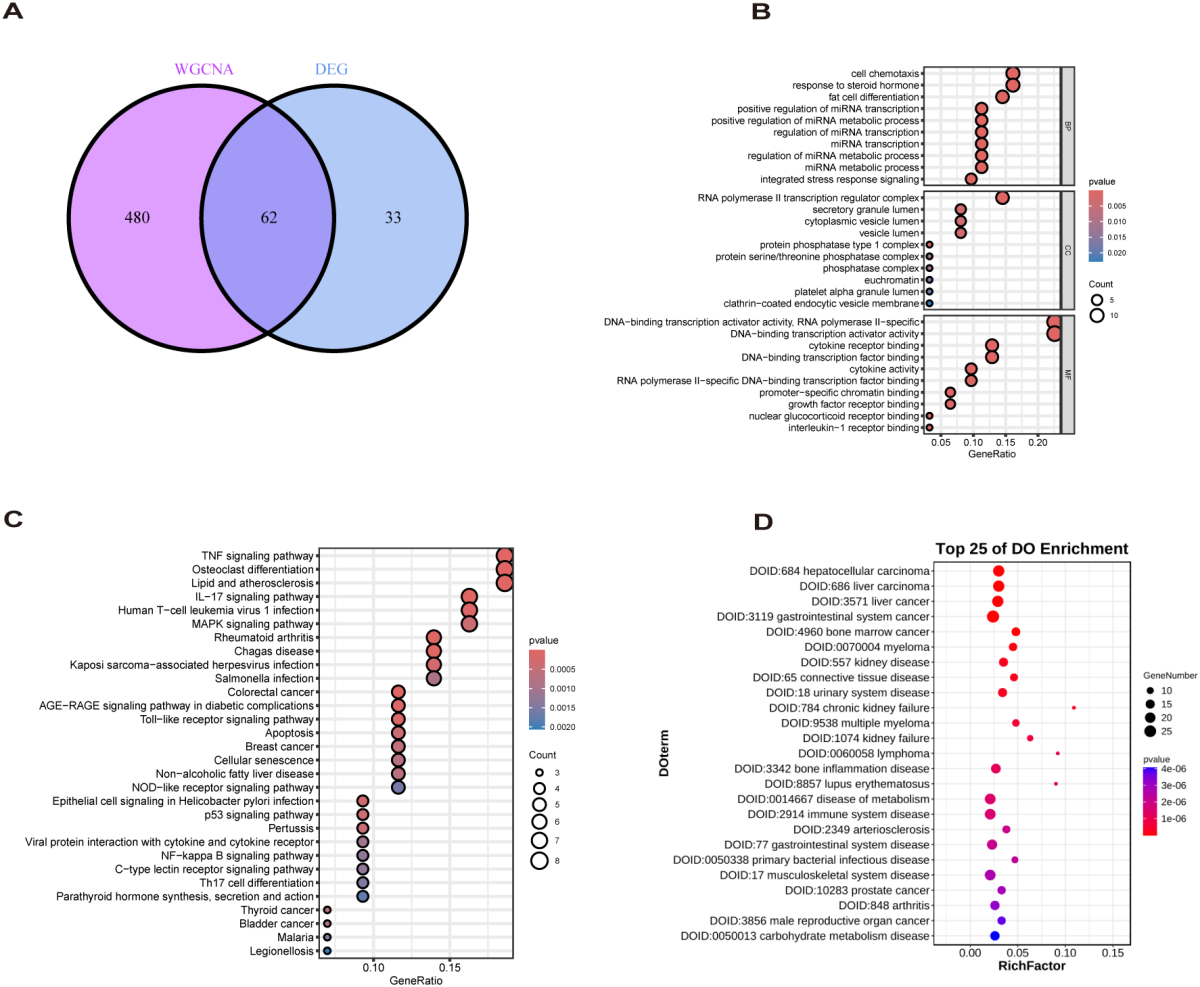
Biological analysis of MASLD-DEGs. **(A)** The Venn diagram shows 62 MASLD-DEGs. **(B–D)** The **(B)** GO, **(C)** KEGG, and **(D)** DO analysis of MASLD-DEGs.

The study design flow chart is shown in **Figure 1**. A total of 49 normal and 93 MASLD patients from GSE24807, GSE33814, GSE89632,GSE63067 and GSE89632 were combined into the training set. Organizations from different platforms showed different patterns of aggregation before the batch effect was removed. Box and PCA plots showed the characteristics of the data distribution before and after the elimination of the batch effect (**Figures 2A, B**). The DEGs between the normal and MASLD groups were then determined using the R package “limma”, based on a p-value < 0.05 and |logFC| ≥ 0.585 as filters. Subsequently, a total of 95 DEGs were identified, of which 55 were down-regulated and 40 were up-regulated (**Figure 2C**). WGCNA was used to screen for modular genes most associated with disease progression. The modules with the dissimilarity < 0.2 were subsequently merged (**Figure 2D**), resulting in a total of 3 modules in this study. A soft-thresholding power (β) of 15 was selected to achieve a scale-free topology fit (R² = 0.9), ensuring the network captured gene expression relationships consistent with scale-free properties(**Figure 2E**). Consequently, these genes were divided into different modules, with the grey color module being positively correlated with MASLD(correlation = 0.29, *P* < 0.001, **Figures 2F, G**). Finally, 62 intersecting genes were generated from two independent methods (**Figure 3A**).

### 3.2 Functional enrichment analysis of MASLD-DEGs

GO enrichment analysis revealed overrepresentation of biological processes including response to steroid hormone, fat cell differentiation, regulation of miRNA metabolic process. Enriched cellular components included RNA polymerase II transcription regulator complex, vesicle lumen and secretory granule lumen. Overrepresented molecular functions comprised cytokine receptor binding, cytokine activity and growth factor receptor binding (**Figure 3B**). KEGG profiling further revealed significant enrichment for TNF signaling pathway, Lipid and atherosclerosis, Non−alcoholic fatty liver disease (**Figure 3C**). DO analysis showed MASLD-DEGs were significantly associated with gastrointestinal system diseases (**Figure 3D**).

### 3.3 Developing a diagnostic model for MASLD-DEGs via machine learning

**Figure 4:**
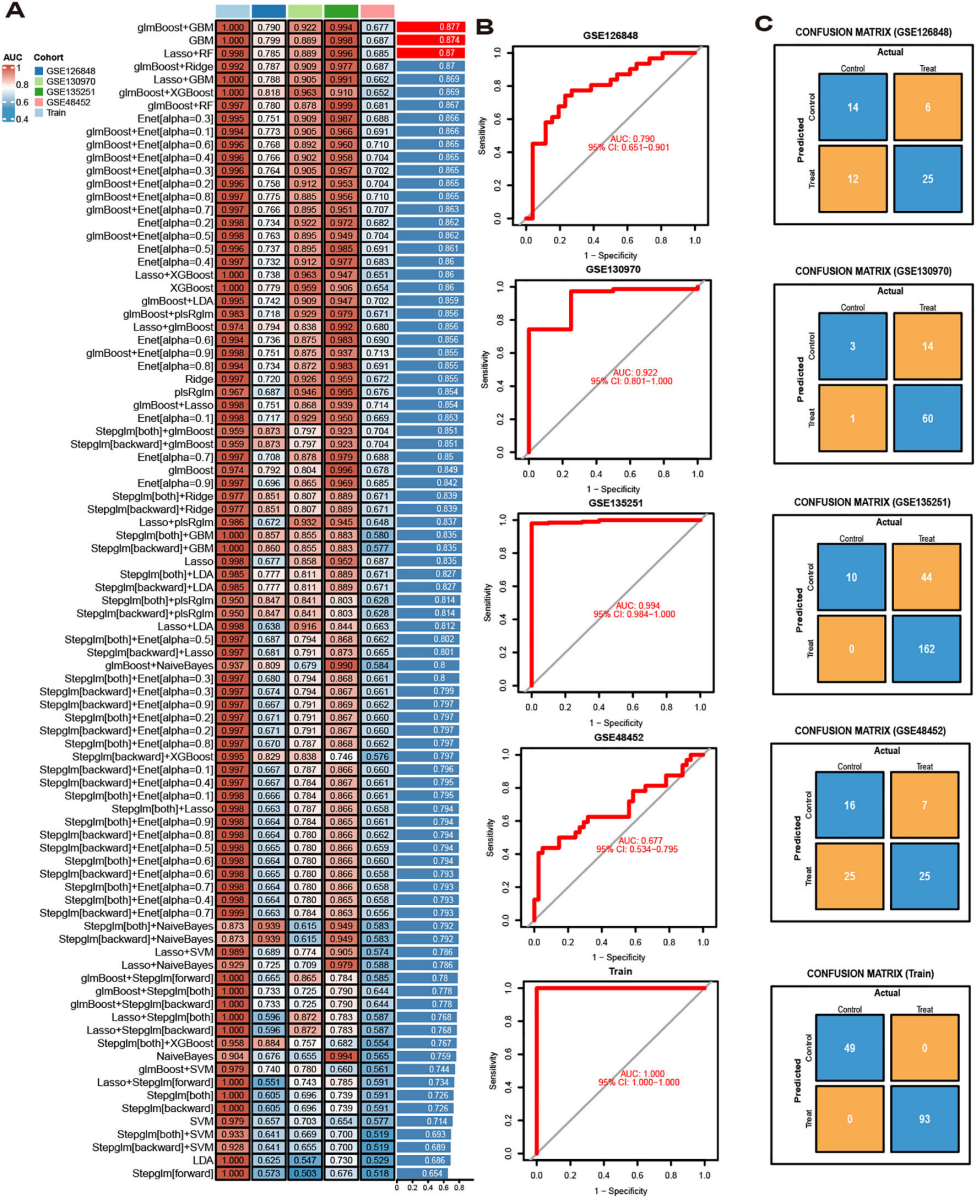
Model Construction. **(A)** ROC curves for 113 algorithms in machine learning. **(B)** ROC curve of the optimal model. **(C)** Confusion matrices for each dataset under the best model.

The diagnostic performance of 12 machine learning algorithms was systematically compared using 10-fold cross-validation, ultimately identifying the most robust model based on 62 MASLD-DEGs. This study constructs predictive models in one training set merged by GSE24807,GSE63067, GSE89632 and GSE33814 and 4 independent validation sets. The best performance was selected by cross-combination of 113 species, and finally the model table constructed by the algorithm of glmBoost+GBM including 17 genes (*IGFBP1*, *ENO3*, *SOCS2*, *GADD45G*, *NR4A2*, *RTP4*, *RAB26*, *CRYAA*, *PPP1R3C*, *MCAM*, *IL6*, *IER3*, *RTP3*, *NR4A1*, *CCL5*, *FOS*, *JUNB*) with average AUC = 0.877 was selected (**Figure 4A**).In the training set, the model showed excellent predictive performance with an AUC of 1.000 and 95% Cl (1.000-1.000). The performance in the four validation sets was as follows: the GSE126848 was 0.790, 95%Cl (0.651-0.901), GSE130970 was 0.922, 95%Cl (0.801-1.000), GSE135251 was 0.994, 95%Cl (0.984-1.000) and GSE48452 was 0.677, 95%Cl (0.535-0.795) (**Figure 4B**). In addition, confusion matrix results showed the difference in model performance on different datasets (**Figure 4C**).

### 3.4 Validation of hub gene expression and their diagnostic value

**Figure 5:**
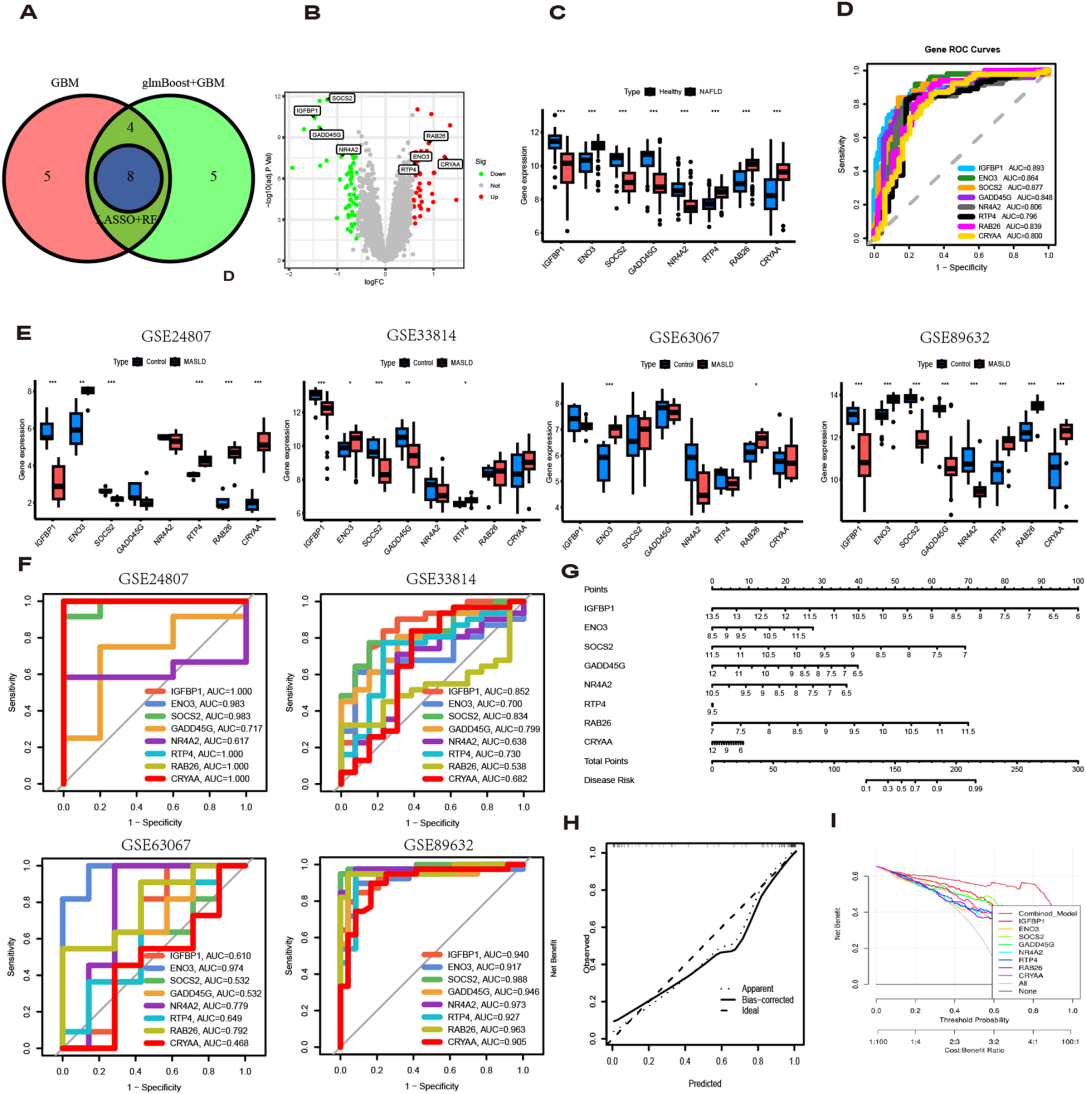
Diagnostic value of eight genes in the optimal model. **(A)** The intersection of the top three machine learning algorithms ranked by average AUC. **(B)** Volcano plot showing the upregulation and downregulation of eight genes. **(C)** The box plot shows the expression of eight genes in the normal group and the MASLD group. **(D)** ROC curves for eight genes. **(E)** Expression levels of 8 genes in different MASLD datasets. **(F)** ROC curves for eight genes in different MASLD datasets. **(G)** Nomogram based on 8 genes. **(H)** Decision curve analysis (DCA) curve. **(I)** Diagnostic models and 8 genes in clinical decision-making (Net Benefit).

To identify key genes, we then take the intersection of the top 3 algorithms identified by machine learning based on their average AUC rankings and finally, 8 intersection genes were identified (*SOCS2*, *IGFBP1*, *GADD45G*, *NR4A2, RAB26*, *ENO3*, *RTP4* and *CRYAA*) (**Figure 5A**). The volcano and boxplots demonstrate the expression of the eight cores. Among them, compared to normal controls, the expression of *SOCS2*, *IGFBP1*, *GADD45G* and *NR4A2* was down-regulated in MASLD, whereas the expression of *RAB26*, *ENO3*, *RTP4* and *CRYAA* was up-regulated in the MASLD(**Figures 5B, C**). QRT-PCR analysis revealed that compared to the control group, mRNA expression of *ENO3* and *CRYAA* was upregulated in the FFAs group, while *IGFBP1* and *SOSC2* were downregulated. *GADD45G*, *NR4A2*, *RAB26*, and *RTP4* showed no statistically significant differences (**Supplementary Figure 1A**). By performing AUC analysis and calculating the ROC value, it was found that *IGFBP1* had the highest diagnostic value in the training set at 0.893, followed by *SOCS2* at 0.877 and ENO3 at 0.864 (**Figure 5D**). Then, we validated again in the four validation sets and found that only one gene, *ENO3*, was up-regulated in the MASLD group compared to the normal control group and all of them showed higher AUC values (**Figures 5E, F**). It is noteworthy that there are inconsistencies between the QRT-PCR results of this study and the bioinformatics analysis. Possible reasons for these discrepancies include: in the FFAs-induced primary hepatocyte model, the primary manifestation is lipid accumulation, while inflammatory injury is not yet significant and does not meet the criteria for MASH. Additionally, the experiments were conducted using mouse primary hepatocytes, whereas the bioinformatics analysis was based on human liver tissue data. Therefore, differences in sample sources and model systems may be the main reasons for the inconsistencies observed in this study. Based on the expression of the 8 hub genes, we plotted a nomogram (**Figure 5G**). In the nomogram, 8 genes correspond to different scores, and their scores are summed to obtain a total score for the different diagnoses of MASLD. The calibration curves demonstrated robust diagnostic reliability of the nomogram for MASLD (**Figure 5H**). Decision curve analysis (DCA) revealed that both the 8 individual genes and their combination provided net benefit (NB) in assessing outcomes of MASLD patients. Notably, the combined nomogram model showed potential to significantly enhance NB compared to individual gene assessments (**Figure 5I**).

### 3.5 SHAP analysis for selecting the optimal predictive gene

**Figure 6:**
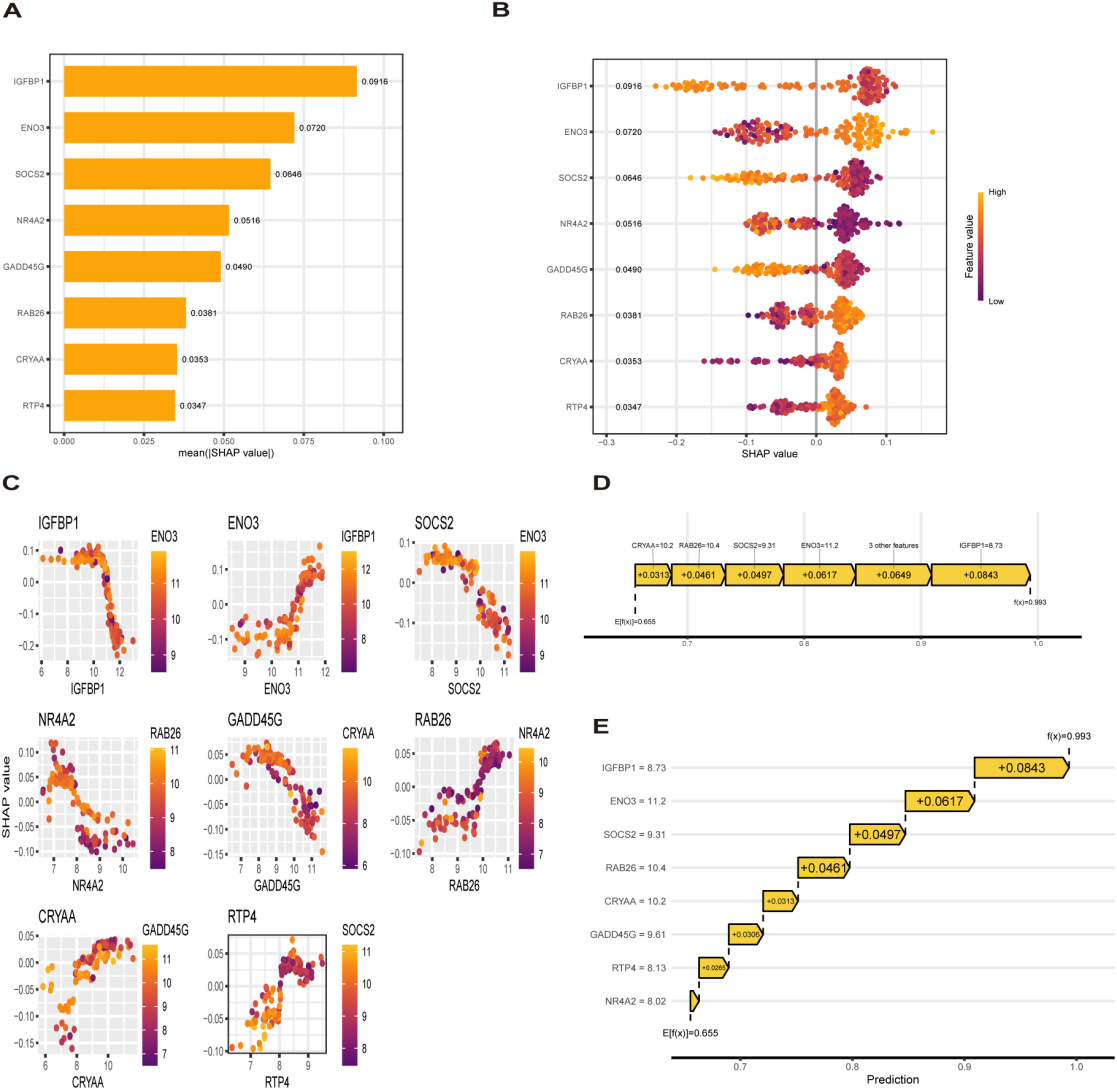
SHAP analysis. **(A, B)** Contribution distribution and direction of 8 genes **(C)** SHAP dependence of 8 genes. **(D, E)** Probability map of 8 genes predicting MASLD.

To explain how machine learning works in predicting MASLD, we use SHAP analysis to elucidate 8 genes. The SHAP summary plot ranking the importance of the feature variables showed that *IGFBP1*, *ENO3*, *SOCS2* were the top three genes with the highest multi-model contribution (**Figure 6A**). The swarm plot is used to show the distribution and direction of the contribution of each characterized gene to the model prediction, from which we can find that higher *IGFBP1* expression is associated with lower MASLD incidence, in contrast to higher *ENO3* eigenvalues and positive SHAP values, which have a stronger impact on the prediction of MASLD incidence (**Figure 6B**). **Figure 6C** illustrate the relationship between SHAP values 2 genes, including *IGFBP1*, *ENO3*, *SOCS2*, *GADD45G*, *NR4A2*, *RTP4*, *RAB26*, *CRYAA*. The predictive analysis, as shown in **Figures 6D, E**, reveals that the model’s performance is primarily influenced by 8 key features. The analysis revealed *IGFBP1* as the most influential factor (0.0843), ahead of *ENO3* (0.0617) and *SOCS2* (0.0497). The strong agreement between the predicted value (f(x) = 0.655) and expected prediction (E[f(x)] = 0.993) indicates excellent model performance. Notably, our analysis pinpoints *IGFBP1*, *ENO3* and *SOCS2* as crucial factors enhancing the model’s predictive power, while providing novel understanding of their biological functions. Numerous literature on *IGFBP1* in MASLD with the limited reports linking *ENO3* to MASLD, suggesting that *ENO3* is a potential therapeutic target. This premise has prompted our focused investigation into the role of ENO3 in MASLD.

### 3.6 Identification of potential drugs

**Figure 7:**
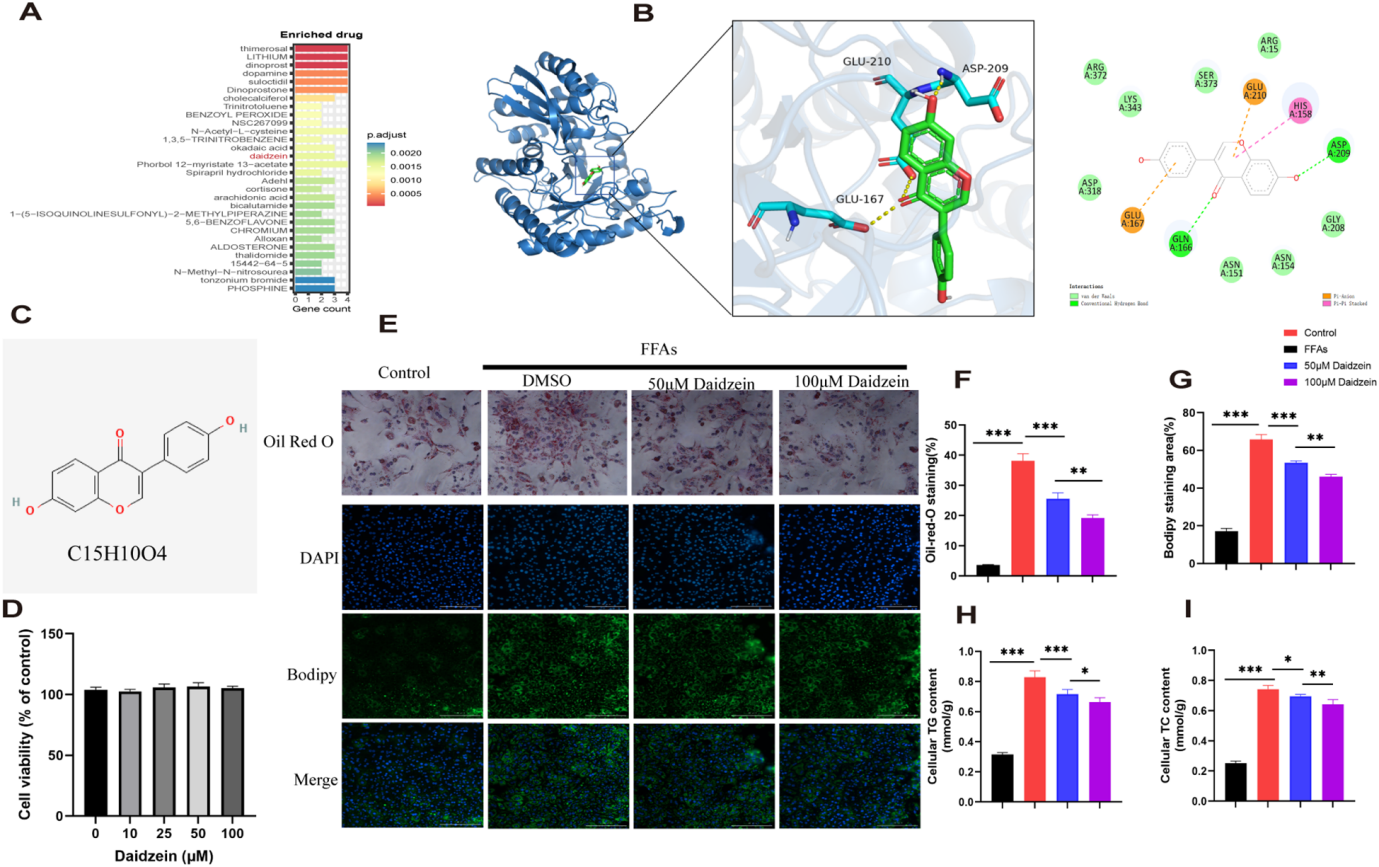
Identification of potential drugs for MASLD. **(A)** The bar plot displays the top 30 candidate drugs. **(B)** Molecular docking analysis of daidzein and *ENO3*. **(C)** Chemical structure of daidzein. **(D)** CCK8 experiment. **(E)** Oil Red O and Bodipy 493/503 staining of primary hepatocytes. **(F, G)** Quantitative analysis by calculating the area of lipid droplets within cells. **(H, I)** Determination of **(H)** intracellular TG and **(I)** TC content. The experiment was repeated three times. * *P* < 0.05, ** *P* < 0.01, *** *P* < 0.001.

genes were analyzed using the DSigDB drug database on Enrichr to identify potential targeting agents. The bar plot displays the top 30 candidate drugs (**Figure 7A**).

Among these compounds, we primarily focused on those related to *ENO3* and identified daidzein as the sole drug candidate (**Supplementary File 1**), which led us to believe that daidzein may be a potential natural compound for treating MASLD. More importantly, daidzein is also a dietary supplement with significant commercial value. According to literature, a binding energy below -4.25 kcal/mol indicates observable interactions, values below -5.0 kcal/mol signify favorable binding, while scores below -7.0 kcal/mol demonstrate strong ligand-receptor binding activity (26). The binding energy score between daidzein and *ENO3* is -7.5 kcal/mol, indicating a strong affinity between the ligand and receptor (**Figure 7B**). Molecular dynamics (MD) simulations were performed to investigate the stability and convergence of the Daidzein + ENO3 complex. As shown in **Supplementary Figure 1B**, the RMSD analysis indicated that the complex system reached equilibrium within 5 ns and subsequently maintained stable fluctuations around 1.4 Å, demonstrating strong binding stability between the small molecule and the target protein. Rg analysis revealed that the complex exhibited relatively stable fluctuations during the simulation, suggesting no significant expansion or contraction occurred in the small molecule-target protein complex throughout the dynamic process (**Supplementary Figure 1C**). The negligible change in SASA further supported that the protein-small molecule complex achieved relatively stable binding (**Supplementary Figure 1D**). Hydrogen bonding plays a critical role in the binding between ligands and proteins. As shown in the **Supplementary Figure 1E**, the number of hydrogen bonds formed between Daidzein and *ENO3* ranges from 0 to 5, with approximately 3 bonds being the most frequent, indicating favorable hydrogen-bond interactions. Additionally, the RMSF values of Daidzein are generally below 2 Å, further suggesting its low conformational flexibility and high stability in the bound state (**Supplementary Figure 1F**). In conclusion, Daidzein exhibits strong binding affinity with *ENO3*. The chemical structure of daidzein is shown in **Figure 7C**. More importantly, we conducted *in vitro* experiments on the pharmacological effects of daidzein. Through the CCK8 experiment, we found that even at a concentration of 100 μM, daidzein had no toxicity to primary hepatocytes (**Figure 7D**). Kim et al. found that 20 or 100 μM daidzein increased insulin-stimulated glucose uptake, while only 100 μM daidzein significantly enhanced basal glucose uptake. Additionally, Liang et al. (27) found that 100 μM daidzein more effectively improved the levels of ALT, AST, IL-1β, IL-6, and TNF-α in LPS-induced primary hepatocytes. Therefore, we subsequently conducted *in vitro* experiments using concentrations of 50 and 100 μM. Oil red and Bodipy 493/503 staining showed that 1mM FFAs stimulation caused lipid droplet accumulation in primary hepatocytes, while daidzein concentration-dependently alleviated lipid accumulation(**Figures 7E–G**). By measuring intracellular TC and TG levels, we further demonstrated that daidzein improved fat deposition in a dose-dependent manner, thereby alleviating MASLD (**Figures 7H, I**).

### 3.7 Daidzein improves MASLD by inhibiting lipid deposition through the ENO3/PPAR signaling pathway

**Figure 8:**
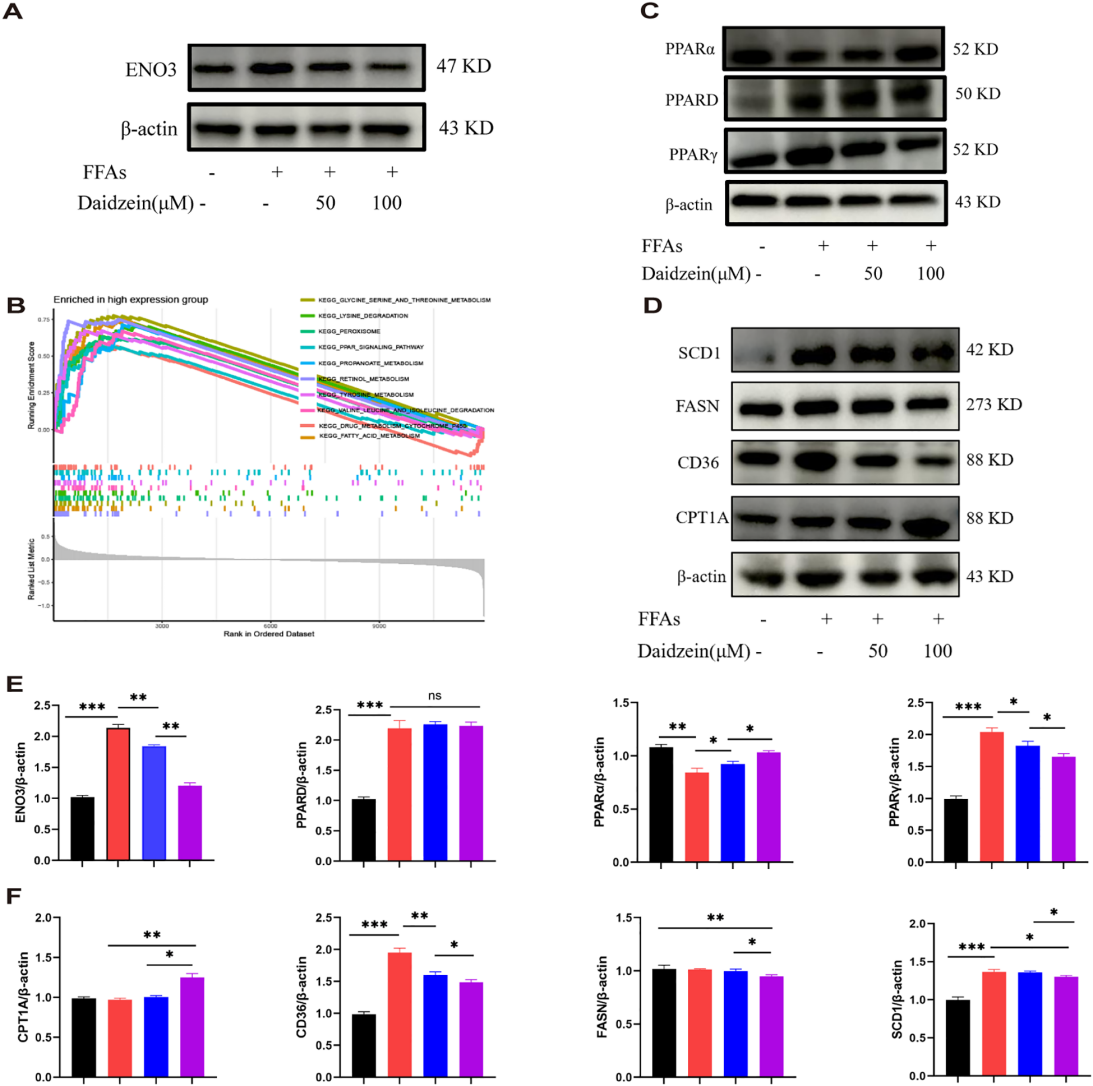
Daidzein alleviates MASLD through the ENO3/PPAR signaling pathway. **(A)** Daidzein reduced the protein expression of *ENO3* in primary hepatocytes. **(B)** GSEA analysis shows the top 10 pathways with high *ENO3* expression. **(C)** Daidzein affects the expression of 3 proteins, PPARα, PPARγ, and PPARD, in primary hepatocytes. **(D)** Daidzein affects the protein expression of downstream proteins (SCD1,FASN, CD36 and CPT1A) in the PPAR pathway. **(E, F)** Perform protein quantification analysis of ENO3, PPAR, PPARγ PPARD, CPT-1A, CD36, FASN and SCD1. The experiment was repeated three times. * *P* < 0.05, ** *P* < 0.01, *** *P* < 0.001.

Western blot analysis revealed that the protein expression of ENO3 was significantly up-regulated in primary hepatocytes stimulated by FFAs compared to the normal group, while daidzein treatment attenuated the expression of ENO3 in a concentration-dependent manner (**Figures 8A, E**). The Gene Set Enrichment Analysis (GSEA) result indicated that pathways such as fatty acid metabolism and the PPAR signaling pathway were significantly enriched in the *ENO3* high-expression group, suggesting that elevated *ENO3* expression may be involved in the regulation of metabolic pathways in MASLD (**Figure 8B**). WB experiments revealed that Daidzein up-regulated PPARα protein expression and down-regulated PPARγ protein expression in FFAs-induced primary hepatocytes, while it had no effect on PPARD protein expression (**Figures 8C, E**). We further validated the expression downstream of the PPAR signaling pathway and found that Daidzein decreased lipid deposition (SCD1, FASN, CD36) protein expression and upregulated fatty acid β-oxidation protein (CPT1A) protein expression (**Figures 8D, F**). In summary, our findings suggest that the ENO3/PPAR signaling pathway maybe a potential mechanism by which daidzein improves fatty degeneration in MASLD.

## 4 Discussion

The prevalence of MASLD is increasing annually and affects 30% of the world’s population. MASLD is also characterized by a progression from early hepatic steatosis-inflammation-fibrosis-cirrhosis-cancer. The launch of Resmetirom fills the gap of nearly 40 years of druglessness in the field of MASH, however, its efficacy still needs to be further improved.

In recent years, advances in high-throughput sequencing, multi-omics integration, and artificial intelligence algorithms have driven a paradigm shift in prognostic research—moving from macroscopic pathological features to molecular mechanisms, from single-omics approaches to multimodal data integration, and from static prediction toward dynamic monitoring (28). Within this context, genome-wide expression profiling offers detailed insights into disease heterogeneity, proving highly valuable for diagnosis, treatment response prediction, and prognosis assessment.

In this study, we conducted biological analysis on transcriptomic data from four MASLD datasets to identify differentially expressed genes (DEGs) between MASLD and control groups. Weighted gene co-expression network analysis (WGCNA) was subsequently applied to pinpoint genes most strongly associated with MASLD progression. By taking the intersection of these gene sets, we ultimately identified 62 MASLD-DEGs. Among 62 MASLD-DEGs, functional enrichment was observed in pathways such as TNF signaling way, Lipid and atherosclerosis and Non-alcoholic fatty liver disease. Subsequently, we used 113 combinations of 12 machine learning algorithms to screen and identify key genes associated with MASLD. The advantage of the integration process lies in the fact that the MASLD model, which is based on multiple machine learning algorithms and their combinations, can achieve stable and consistent performance, thereby significantly improving the specificity and sensitivity of key gene detection. Ultimately, glmBoost combined GBM was identified as the optimal model, and the specificity and sensitivity of the model were further validated using the validation set. However, we found that the model performed poorly on the GSE48452 dataset (AUC = 0.677), which may be related to database sources, platform differences, and sample processing.

SHAP analysis, a tool considered the “gold standard” in the field of machine learning interpretability, further helps us identify the genes most closely associated with the progression of MASLD. Through SHAP analysis, we found that 3 genes:Insulin Like Growth Factor Binding Protein 1 (*IGFBP1)*, Enolase 3 (*ENO3), and* Suppressor of cytokine signaling 2 (*SOCS2)* contributed the most to the model. Numerous studies have shown that *IGFBP1* is negatively correlated with the progression of MASLD (29, 30). The protein encoded by *ENO3*, the beta (β) enolase subunit, has a broad tissue distribution and is found in the liver, lungs, bones, and heart, among others (31). There are few studies on the correlation between *ENO3* and MASLD. Liu et al. (32) found that the expression of *ENO3* was positively correlated with the severity of MASLD and validated this finding in mice with MASLD induced by a high-fat diet. In addition, Lu et al. (33) found that *ENO3* inhibits ferroptosis by upregulating *GPX4* expression and enhancing lipid accumulation, thereby mediating the progression of MASH. The above evidence demonstrates the potential of *ENO3* as a novel biomarker for MASLD. Further research is needed to understand how *ENO3* drives the progression of MASLD. *SOCS2* is one of the classic molecules of cytokine signaling and has recently been found to have anti-inflammatory effects (34). The “multiple hit” theory is the mainstream pathogenesis of MASH, in which inflammation plays an important role in the progression of MASH. Inflammation is not only a hallmark that distinguishes MASH from simple fatty liver disease, but also a key driver of disease progression to cirrhosis and hepatocellular carcinoma (35). Li et al (34). found that overexpression of *SOCS2* in macrophages inhibited the development of MASH *in vivo*. Yu et al. (36) also found that *SOSC2* may be a key gene for predicting the progression of MASLD. Given the heterogeneity of MASLD, there are currently few drugs available for its treatment. Diet and exercise control remain the preferred treatment options recommended by many guidelines and clinicians (37). Therefore, researching and exploring MASH intervention drugs and targets to prevent the occurrence of cirrhosis and liver cancer is a major scientific issue that needs to be addressed in the field of life sciences. Therefore, targeting essential genes using bioinformatics methods is expected to significantly improve drug discovery efficiency and reduce costs. Using the DSigDB database, this study identified anti-MASLD drugs by connecting the eight genes that construct the MASLD model. Here, we focused on drugs targeting the *ENO3* and ultimately discovered the drug Daidzein. Traditional Chinese medicine has a history of thousands of years in the treatment of chronic liver disease. Through dialectical treatment and a holistic approach, clinical and basic research on the use of traditional Chinese medicine to treat MASLD continues to emerge, demonstrating promising prospects for application. Daidzein is a major isoflavone compound found primarily in legumes such as soybeans and kudzu, as well as in grasses and grains (38). Research has found that daidzein has protective effects against a variety of diseases, including breast cancer, prostate cancer, diabetes (39), and cardiovascular disease (40). A cross-sectional study from NHANES 2017–2018 showed that daidzein intake was negatively associated with the incidence of MASLD (41). Kim et al. (42) found that dietary supplements containing ≥ 0.5 g/kg of daidzein improved MASLD by promoting fatty acid β-oxidation and mRNA levels of adiponectin and leptin-related genes. *In vitro* studies revealed that daidzein dose-dependently ameliorated hepatic steatosis in primary hepatocytes. These findings provide a novel therapeutic strategy for the treatment of MASLD. GSEA analysis revealed that high expression of *ENO3* correlates with the PPAR signaling pathway. Therefore, we examined the protein expression of PPAR and its downstream molecules. PPARs are a class of nuclear receptors that can bind to various endogenous or exogenous lipophilic ligands, such as fatty acids, fatty acid derivatives, and anti-diabetic drugs. This binding regulates gene expression and transcription, influencing a variety of physiological and pathological processes including lipid metabolism, glucose metabolism, inflammatory responses, cell differentiation, proliferation, and apoptosis (43). PPAR comprises three subtypes—*PPARα, PPARδ, and PPARγ*—each with distinct tissue distribution and functions (44). *PPARα* is predominantly expressed in the liver, heart, and skeletal muscle, where it regulates fatty acid oxidation, ketogenesis, energy homeostasis, and lipid metabolism, offering protection against cardiovascular diseases and hyperlipidemia (45). Carnitine palmitoyltransferase-1a(*CPT-1A*) catalyzes the conjugation of long-chain fatty acyl coenzymes with L-carnitine to form fatty acylcarnitine, enabling its transport across the inner mitochondrial membrane into mitochondria for β-oxidation. This process represents a critical regulatory point in the fatty acid β-oxidation pathway. Research indicates that *PPARα* can enhance *CPT-1A* expression and activity, thereby increasing the capacity for fatty acids to enter mitochondria for oxidative metabolism and improving overall fatty acid oxidation (46). In contrast, *PPARγ* is mainly found in adipose tissue, intestine, and immune cells. It promotes fatty acid synthesis and storage, enhances insulin sensitivity, and modulates inflammatory responses, making it a key target in the treatment of diabetes and metabolic syndrome (47). Cluster of differentiation 36 (*CD36*) is a fatty acid transporter and a target gene of *PPARγ (* 48). *CD36* promotes the uptake of free fatty acids (FFAs) by hepatocytes, increasing intracellular lipid accumulation and leading to hepatic steatosis. Stearyl‐coenzyme A desaturase 1 (*SCD-1*) and fatty acid synthase (*FASN)* are key enzymes for *de novo* fatty acid synthesis in the liver, while also functioning as downstream molecules of *PPARγ (* 49). Research indicates that hepatic *SCD1* deficiency reduces hepatic TG accumulation, increases fatty acid oxidation, and decreases *de novo* TG synthesis (50). Activation of hepatic *PPARγ* expression can upregulate the expression of lipid synthesis genes such as *FASN*, thereby accelerating the progression of MASLD (51). This study found that Daidzein ameliorates MASLD by promoting PPARα and CPT-1A expression to enhance fatty acid β-oxidation on one hand, and by suppressing PPARγ, FASN, SCD1 and CD36 expression to inhibit lipid synthesis on the other hand, independently of PPARδ.

However, this study also has several limitations. First, although multiple GEO datasets were included, the sample size remains limited and the constructed model lacks validation with large-sample real-world data. Second, the mechanism by which *ENO3* influences MASLD lipid metabolism processes requires further investigation. Third, the efficacy of Daidzein requires further validation through *in vivo* experiments, and its mechanisms of action have only been preliminarily explored.

## 5 Conclusion

8 genes (*IGFBP1*, *ENO3*, *SOCS2*, *GADD45G*, *NR4A2*, *RTP4*, *RAB26*, *CRYAA*) were identified based on biological information from machine learning. SHAP analysis further focused on 3 key genes, *IGFBP1*, *ENO3* and *SOSC2*. Importantly, we also identified Daidzein, a potential natural drug against MASLD. Molecular docking and molecular dynamics mimetics showed that Daidzein docked well with *ENO3*, and further *in vitro* experiments indicated that Daidzein may ameliorate MASLD through the PPAR/ENO3 signaling pathway.

## Data Availability

The datasets presented in this study can be found in online repositories. The names of the repository/repositories and accession number(s) can be found in the article/**Supplementary Material**.

## Supplementary material

Supplementary Figure 1 Molecular dynamics simulation analysis. **(A–E) (A)** The mRNA expression levels of 8 genes. **(B)** RMSD, **(C)** Rg, **(D)** SASA, **(E)** HydrogenBond number, and **(F)** RMSF analysis of soybean glycoside and ENO3.
