# Supplementary figures and images for: Development of a diagnostic model for MASLD and identification of daidzein as the potential drug using bioinformatics analysis and experiments
Source: Front Immunol. 2025 Oct 22;16:1698740. doi: 10.3389/fimmu.2025.1698740 (PMC12586024; doi:10.3389/fimmu.2025.1698740)

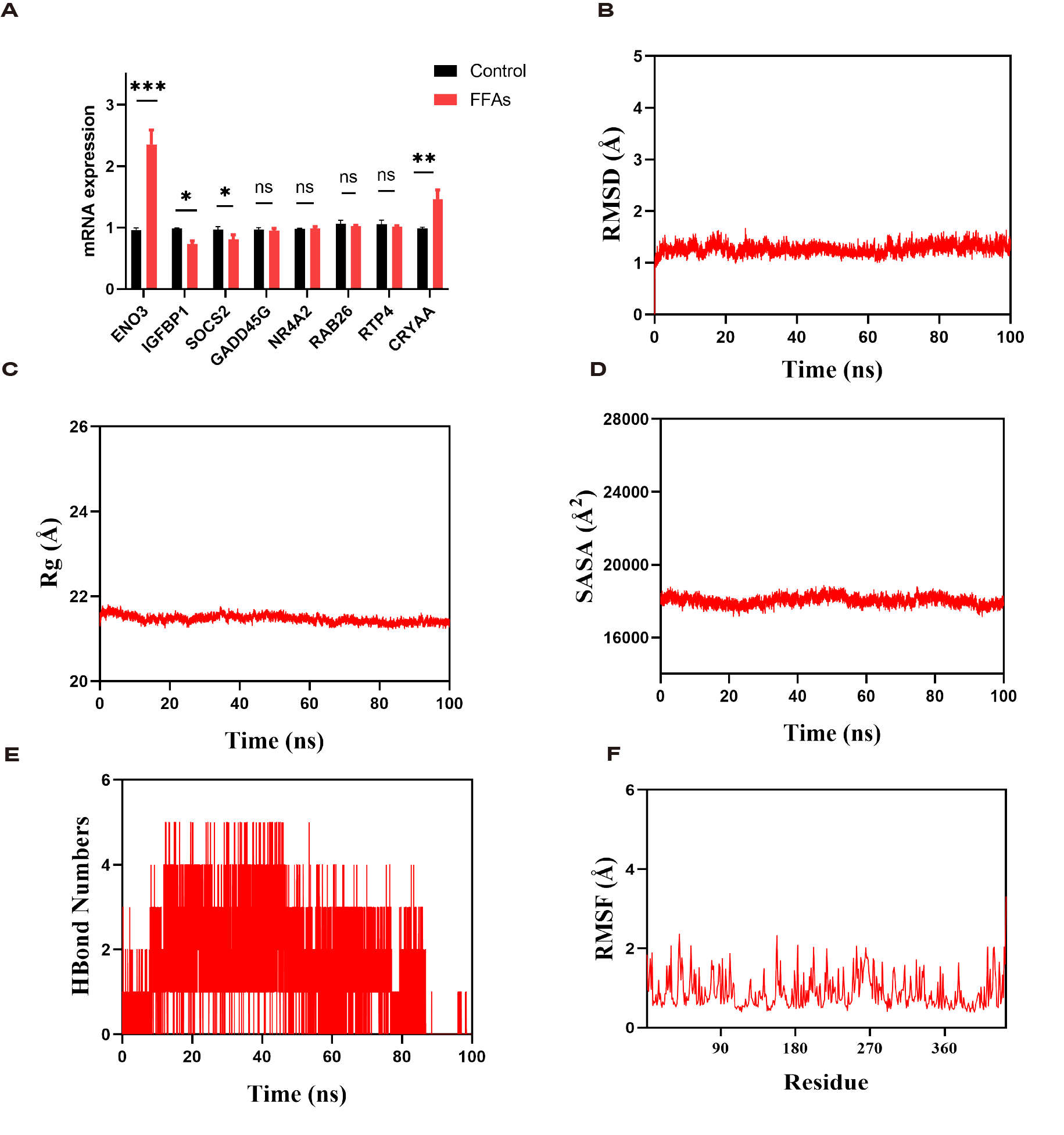

Supplement: Supplementary Figure 1 — Molecular dynamics simulation analysis. (A–E) (A) The mRNA expression levels of 8 genes. (B) RMSD, (C) Rg, (D) SASA, (E) HydrogenBond number, and (F) RMSF analysis of soybean glycoside and ENO3. [file Image1.tif]
